# A novel long non-coding RNA-KAT7 is low expressed in colorectal cancer and acts as a tumor suppressor

**DOI:** 10.1186/s12935-019-0760-y

**Published:** 2019-02-26

**Authors:** Qingmei Wang, Rongzhang He, Tan Tan, Jia Li, Zheng Hu, Weihao Luo, Lili Duan, Wenna Luo, Dixian Luo

**Affiliations:** 1 Translational Medicine Institute, National & Local Joint Engineering Laboratory for High-throughput Molecular Diagnosis Technology, The First People’s Hospital of Chenzhou, University of South China, Chenzhou, 423000 People’s Republic of China; 2 Center for Clinical Pathology, The First People’s Hospital of Chenzhou, University of South China, Chenzhou, 423000 People’s Republic of China; 3 Department of Clinical Pharmacology, Xiangya Hospital, Central South University, Changsha, 410078 People’s Republic of China

**Keywords:** lncRNA-KAT7, Colorectal cancer, Proliferation, Invasion, Migration, Tumor suppressor

## Abstract

**Background:**

The abnormal expression of many long non-coding RNAs (lncRNAs) has been reported in the progression of various tumors. However, the potential biological roles and regulatory mechanisms of long non-coding RNAs in the development of colorectal cancer (CRC) have not yet been fully elucidated. Therefore, it is crucial to identify that lncRNAs can be used for the clinical prevention and treatment of CRC.

**Methods:**

In our previous work, we identificated a novel lncRNA, lncRNA-KAT7, and found that the expression of lncRNA-KAT7 in CRC tissues was significantly lower than that in matched normal intestinal tissues, and the expression in CRC cell lines was lower than that of normal intestinal epithelial cells (P < 0.05). Besides, the expression of lncRNA-KAT7 is negative associated with age, tumor size, tumor differentiation, lymph node metastasis of CRC patients. The potential biological effects and molecular mechanisms of lncRNA-KAT7 in CRC were evaluated using a series of CCK-8 assay, clone formation assay, EdU proliferation assay, scratch determination, transwell determination, western blot analysis, and nude subcutaneous tumorigenesis model construction cell and animal experiments.

**Results:**

The expression of lncRNA-KAT7 in CRC tissues was lower than that in matched normal tissues and normal intestinal epithelial cells (*P *< 0.05). Decreased expression of lncRNA-KAT7 is associated with clinicopathological features of poor CRC patients. In vitro experiments showed that up-regulation of lncRNA-KAT7 expression in CRC cells inhibited cell proliferation and migration. In vivo animal experiments showed that the lncRNA-KAT7 also inhibited tumor growth. Western blot analysis showed that the expression of lncRNA-KAT7 was up-regulated in HCT116 cells, the expression of E-cadherin increased, and the expression of Vimentin, MMP-2 and β-catenin protein was down-regulated so did the phosphorylation NF-κB P65. The results confirm that the expression of lncRAN-KAT7 can inhibit the malignant phenotype of CRC cells.

**Conclusions:**

Up to now, as a novel lncRNA, lncRNA-KAT7 has not any relevant research and reports. The results confirm that the expression of lncRNA-KAT7 can inhibit the malignant phenotype of CRC cells. And it can be used as a new diagnostic biomarker and therapeutic target for the development of CRC.

## Background

Colorectal cancer (CRC) is the leading cause of cancer mortality in all humans, with approximately 65% of 5-year survival rate of CRC patients [1]. In recent years, the incidence of CRC is on the rise, showing a trend of younger age. It is still the focus of global health concerns [2]. The specific pathogenesis for CRC remains unclear. It has been reported that many oncogenes and tumor suppressors are involved in the development of CRC [3]. In the past decade, although the popularity of early CRC screening and the continuous advancement of CRC diagnosis and treatment, the mortality rate of CRC has declined [4]. However, the mortality rate for patients with CRC metastasis remains high, and metastasis is still one of the key factors causing low clinical efficacy, poor survival period, and poor prognosis in CRC patients [5]. Therefore, the prevalence and poor outcome of CRC prompted us to reveal the pathological mechanism of CRC progression and to find effective biomarkers for diagnosis and prognosis.

In recent years, a large amount of evidence suggests that long non-coding RNA (lncRNA) has become an indispensable participant in the development of different human tumors [6]. Most of human genome is transcribed into RNA, but only about 2% of RNA encodes proteins. RNAs lacking protein-coding capacity and open reading frame (ORF) are referred to as non-coding RNAs (ncRNAs) and are divided into small ncRNAs and long ncRNAs (lncRNAs) groups [7, 8]. More and more lncRNAs have been found to participate in different physiological and pathological processes in the body, but the vast majority of their functions are still unclear [9]. LncRNAs are non-coding transcripts of 200 nucleotides or more, and most of which are located in nuclear. They have no ORF that results in the loss of translation capacity and emerges as essential regulators of cell growth and tumor metastasis [10]. Many lncRNAs are expressed at low levels and show tissue and cell type-specific expression patterns, whereas the abnormal expression of lncRNAs is attributed to the pathogenesis of some malignant tumors including CRC [11]. It has been reported in the literature that lncRNA has been identified as an oncogene, anti-oncogene and prognostic predictor [12].

Only a few discovered lncRNAs play a key role in different biological processes [13]. The abnormal expression of multiple lncRNAs is associated with CRC, suggesting that lncRNA plays an important role in the development of CRC. Some lncRNA, such as H19, HOTAIR, MALAT1, CCAT2 expressed highly in CRC, are responsible for cell proliferation, migration, and invasion of CRC [14]. In addition, Ye et al. [15] found that a novel lnc-GNAT1-1 is low-expressed in CRC and acts as a tumor suppressor by regulating the RKIP-NF-κB-Snail pathway. Miao et al. [16] found that FOXF1-AS1 affects tumor metastasis by regulating the expression of E-cadherin and Vimentin in non-small cell lung cancer. Recently, lncRNA has become one of the research hotspots, and lncRNA plays a role in oncogenes and tumor suppressor genes in various biological processes such as epigenetic regulation, transcription and translation regulation, splicing, imprinting, cell development, metastasis, and apoptosis [17]. One of the most critical steps in the tumor cell metastasis cascade is to gain invasive capabilities, including disrupting cell–cell connections, degrading the cell matrix, and activating pathways that control the cytoskeleton dynamics. EMT is a key factor in biological processes. Epithelial cells lose their polarity and convert to a mesenchymal phenotype that causes the metastasis of cancer cells [17–21]. EMT enhances the invasion of cancer cells, in response to environmental triggers, strengthens invasive functions, and also conduces to cell growth and survival [22]. In addition, the literature also confirms that many lncRNAs promote or inhibit the occurrence and progression of tumors by modulating the EMT pathway [23].

**Fig. 1:**
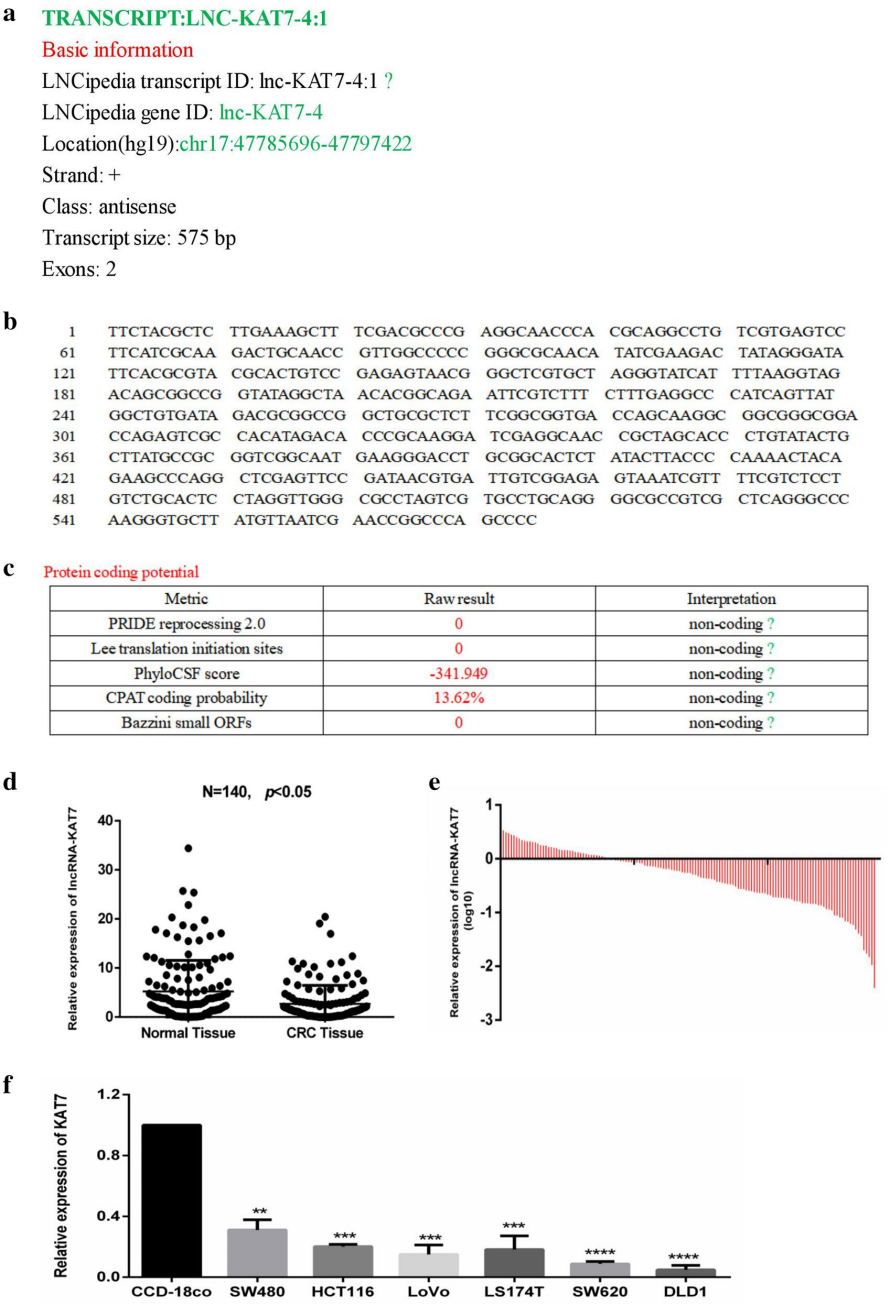
The sequence characteristics of lncRNA-KAT7 and its relative expression level in CRC. **a** Basic information of lncRNA-KAT7; **b** complete sequence of lncRNA-KAT7; **c** the prediction of protein-coding ability of lncRNA-KAT7; **d** expression of lncRNA-KAT7 was decreased in tumor tissues than paired adjacent normal colorectal tissues (P < 0.05); **e** expression of lncRNA-KAT7 was significantly down-regulated in tumor tissues compared to the paired adjacent normal colorectal tissues (log10, P < 0.001); **f** Relative expression of lncRNA-KAT7 in six CRC cell lines (HCT116, SW620, LoVo SW480, DLD1and LS174T) and normal human colon tissue cells (CCD-18Co).*P < 0.05, Two-side Student’s *t* test; n = 3

To investigate the role of lncRNAs in CRC, we performed lncRNA microarray assays using “Agilent All-Human Genome Oligonucleotide Microarray (4 × 44 K)” (Bohai Biotechnology, Shanghai, China) according to the standard protocol of the expression profiling microarray. Three pairs of CRC normal tissues and tumor tissues were tested. It was found that lncRNA-KAT7 was significantly lower expressed in CRC tumor tissues than that in normal tissues compared with normal intestinal epithelial tissues in differentially expressed lncRNAs. KAT7 is an lncRNA located on the human chromosome 17, plus the hg19 region, which contains 575 transcripts without 5′ cap structure3′ polyadenylation tail (Fig. 1a, b), Kozak sequence or ORF, and with a PhyloSCF score of 342 (Fig. 1c). Biopredictive software suggests that lncRNA-KAT7 does not have protein coding capability. LncRNA-KAT7 was significantly decreased in CRC tumor tissues, indicating that lncRNA-KAT7 may be involved in tumorigenesis and progression of CRC.

To the best of our knowledge, there is no relevant reports on lncRNA-KAT7 in CRC. Therefore, the purpose of this study was to determine the expression and biological effects of lncRNA-KAT7 in CRC in the cellular, animal level and human specimens, especially its role in the metastasis of CRC tumors. This study provides important clues for finding new CRC biomarkers and preventing and treating targets.

## Materials and methods

### Patients and samples

This study included 140 patients with CRC diagnosed in the First People’s Hospital of Chenzhou City between 2014 and 2016. Fresh colorectal neoplasms and matching normal tissues (located > 2 cm away from the tumor boundary) were obtained from 140 patients, and samples should be placed in liquid nitrogen quickly and stored frozen until RNA extraction. All specimens were examined histopathologically and no other treatment had been performed before surgical resection. The clinical characteristics of the patients are shown in Table 1. All experiments in this study were conducted in accordance with guidelines and procedures.

**Table 1:** Relationship between KAT7 and clinicopathological characteristics in patients with CRC (N = 140; *χ*^*2*^test)

Clinicopathological characteristic	Total	LncRNA-KAT7 expression		*χ*^*2*^ value	*P* value
		High (%)	Low (%)		
Age (years)				0.732	0.392
< 60	59	27 (45.8)	32 (54.2)		
≥ 60	81	43 (53.1)	38 (46.9)		
Sex				1.036	0.309
Male	76	41 (53.9)	35 (46.1)		
Female	64	29 (45.3)	35 (54.7)		
Tumor site				4.877	0.027
Rectum	77	45 (58.4)	32 (41.6)		
Colon	63	25 (39.7)	38 (60.3)		
Tumor size				6.494	0.011
< 5 cm	77	46 (59.7)	31 (40.3)		
≥ 5 cm	63	24 (38.1)	39 (61.9)		
Tumor differentiation				4.516	0.034
Well/moderate	124	66 (53.2)	58 (46.8)		
Poor	16	4 (25)	12 (75)		
Depth of invasion				0.365	0.546
T1 + T2	12	7 (58.3)	5 (41.7)		
T3 + T4	128	63 (49.2)	65 (50.8)		
Lymph node metastasis				4.118	0.042
N0	72	42 (58.3)	30 (41.7)		
N1/2	68	28 (41.2)	40 (58.8)		
TNM stage				1.830	0.176
I + II	68	38 (55.9)	30 (44.1)		
III + IV	72	32 (44.4)	40 (55.6)		
Distant metastasis				0.504	0.478
Absence	119	61 (51.3)	58 (48.7)		
Presence	21	9 (42.9)	12 (57.1)		

### Cell lines and cell culture

The normal human colonic tissue cell CCD-18Co and human CRC cell lines HCT116, SW620 were purchased from the Institute of Cell Biology of the Chinese Academy of Sciences (Shanghai, China), human CRC cell lines LoVo and LS174T were donated by Dalian Medical University, and Human CRC cell lines SW480 and DLD1 were donated by Sun Yat-Sen Cancer Center. The cell lines were subcultured and preserved in our lab. The cells were cultured in RPMI 1640 or DMEM (Gibco) medium supplemented with 10% fetal bovine serum (FBS, Gibco) and 1% double antibiotics (penicillin, streptomycin) at 37 °C and under 5% CO_2_ in a cell incubator.

### RNA extraction and quantitative real-time PCR

Total RNA was extracted from each cell sample and fresh frozen tissues using Trizol reagent (Life Technologies, Carlsbad, CA, USA) according to the manufacturer’s instructions. The absorbance ratio at 260/280 nm of the isolated RNA were measured with NanoDrop 2000c spectrophotometer (Thermo Fisher). For lncRNA quantification, GAPDH was used as internal control, and GoScript™ RT System (Promega, USA) were used for quantitative real time reverse transcript PCR (qRT-PCR). The primer sequences were as follows: lncRNA-KAT7 forward: 5′-AGCTCTTGGTTGAGCCCTTC-3′, lncRNA-KAT7 reverse: 5′-GGGGCTGTGTGTGATTTTGTC-3′; GAPDH forward: 5′-ACCACAGTCCATGCCATCAC-3′, GAPDH reverse: 5′-TCCACCCTGTTGCTGTA-3′; Axin2 forward: 5′-AGCCAAAGCGATCTACAAAAGG-3′, Axin2 reverse 5′-AAGTCAAAAACATCTGGTAGGCA-3′.

Each experiment was performed in triplicate. The fold change for each gene relative to the control group was determined using the 2-ΔΔCT method.

### Vector construction and cell stable transfection

In order to study the biological effect of lncRNA-KAT7 in CRC, the cDNA sequence of human full-length lncRNA-KAT7 was introduced into the empty vector pcDNA3.1 to construct a recombinant plasmid pcDNA-KAT7. HCT116 and DLD1 cells were then transiently transfected with pcDNA-KAT7 using Lipofectamine^®^2000 Reagent (Invitrogen) according to the manufacturer’s instructions. 24 h later, different concentrations of neomycin (G418) were added to the medium of HCT116, DLD1 cells to screen the cells transfected stably with pcDNA-KAT7. Blank control cells and the cells with lncRNA-KAT7 over-expression were named as control, HCT116/pcDNA-3.1, DLD1/pcDNA-3.1, HCT116/pcDNA-KAT7, DLD1/pcDNA-KAT7, respectively.

### Cell proliferation assay

Log phase cells (5 × 10^3^ cells per well) were seeded in 96-well plates. The cell proliferation was assessed with Cell Counting Kit-8 (CCK8) according to the manufacturer’s instructions. Proliferation rates were determined at 0, 24, 48, 72 and 96 h after transfection by measuring the absorbance. The Cells in each group were tested for 5 replicates. The EDU Cell Proliferation Assay Kit (Green Sky, Shanghai, China) was used to evaluate cell proliferation. At the log phase of cell growth, the cells were spiked with 5-ethynyl-29-deoxyuridine (EdU) according to the manufacturer’s instructions. Briefly, the cells were incubated with 50 mM EdU for 3 h, and then the cellular nuclei were treated with DAPI staining (Sigma) at a concentration of 1 mg/ml for 10 min, the cell proliferation indexes were photographed and recorded by fluorescence microscopy.

### Clone formation assay

Cells in the growth phase (300 cells per well) were seeded in 6-well plates, and cultured in cell incubator at 37 °C in 5% CO_2_ for about 10 days until large colonies were visible. Colony formation was determined by counting the number of visible colonies after being fixed with 4% paraformaldehyde for 15 min and stained with crystal violet dye for 10 min.

### Transwell assay

Cell migration and invasion were measured using a Corning polycarbonate film insert transwell chamber (Corning, USA) containing 8 μm pores in the presence or absence of Matrigel (BD Biosciences, USA) according to the manufacturer’s protocol. Cells (5 × 10^4^ cells per well) were seeded in the upper of chamber for migration assay (without Martrigel) for 24 h, and cells (10 × 10^4^cells per well) were seeded in the upper of chamber for invasion assay (use Martrigel) for 24 h. The medium containing 10% FBS was placed in the lower chamber as an attractant. 24 h later, the cells were fixed in 4% paraformaldehyde for 20 min and then stained with 0.1% crystal violet for 10 min. The cells in the upper chamber were removed with a cotton swab and dried, and the images were captured in 6 random zones under each microscope. The number of cells was counted by Image software. The mean cell count of three independent membranes was defined as the migration or invasion index. The experiment was repeated three times.

### Wound healing assay

Cells were cultured in 24-well plate until the cell density reached 80–90% confluence. Then, two vertical wound were scratched using a 100 μl micro tip. The cells were washed twice with phosphate buffered saline (PBS) to remove cell debris and then the cells were cultured in serum-free medium. The images were captured at 0 h, 24 h and 48 h, and the width of the wound was assessed by using Images-Pro Plus software.

### Nude subcutaneous tumorigenesis model

6-week-old male athymic BALB/c nude mice were maintained in laminar flow cabinets under specific pathogenic conditions. All animal work was conducted in accordance with national guidelines. All animal experiments were approved by the Ethical Committee of the First People’s Hospital of Chenzhou City, university of South China. Stably transfected cells were washed twice and resuspended in 1 × phosphate buffer. 100 μl of DLD1-pcDNA-3.1 and DLD1-pcDNA-KAT7 (3 × 10^6^ cells per mouse) cells suspension were injected subcutaneously into the back of BALB/c athymic mice (n = 6 per group). The size of the tumor was recorded every week with vernier caliper, and the primary tumor growth was calculated from the formula (length × width^2^)/2 [24]. Four weeks after the injection, the nude mice were sacrificed, and the dorsal tumors were excised and weighed after they had been photographed and measured. Sections from the tumors were stained with hematoxylin and eosin (H & E) for general histology, or immunostained with specific antibodies (anti-Ki67 for proliferation) [25].

### Western blot analysis

The expression levels of E-Cadherin, Vimentin, MMP-2, NF-κBp65, p- NF-κBp65, β-catenin, Snail, Twist and ZEB1proteins were detected by western blot. The total protein was extracted and the protein concentration was measured by BCA method. 40ug proteins were subjected to SDS–polyacrylamide gel electrophoresis (SDS-PAGE) and then transferred to polyvinylidene difluoride membranes (PVDF). The membranes were incubated with primary antibodies followed by horseradish peroxidase(HRP)-labeled secondary antibody. Protein bands were detected using the enhanced chemiluminescence with imaging system (Bio-Rad, CA, USA). We used β-actin as a loading control.

### Statistical analysis

All data are based on the mean ± standard deviation (SD) from at least three independent experiments. Statistical analysis was performed using SPSS 18.0 software and Graph Pad Prism 6. The relationship between clinicopathological features and lnc-KAT7 expression level was assessed using Chi squared test (χ^2^ test). One-way analysis of variance (ANOVA), paired *t* test and unpaired *t* test were used for statistical comparison. All *P* values were two-sided, and *P* values < 0.05 were considered significant.

## Results

### Basic information of lncRNA-KAT7 gene

As described above, we previously performed lncRNA expression microarray analysis using the “Agilent Whole Human Genome Oligonucleotide Microarray (4 × 44 K)” according to a standard protocol to find differential expression lncRNA between CRC tissue and normal colon tissue. A novel lncRNA, lncRNA-KAT7 was screened from the differentially expressed lncRNA transcripts. LncRNA-KAT7 (ENST00000512720.1) is located on the positive strand of hg19 region of human chromosome 17, and the transcript length is 575 base pairs. Bioinformatics software predicts that there is no open reading frame (ORF) and the PhyloSCF score is -342, suggesting that there is no protein coding ability, 5′ cap structure or 3′ polyA tail of lncRNA-KAT7 (Fig. 1a–c).

### LncRNA-KAT7 is low expressed in CRC tissues

The relative expression levels of lncRNA-KAT7 were measured using qRT-PCR in 140 patients with CRC, normalized to GAPDH. LncRNA-KAT7 was down-regulated in 71.4% (100/140) of CRC tissues compared with matched adjacent normal tissues (*P *< 0.05, Fig. 1d, e). We then evaluated whether lncRNA-KAT7 expression was associated with any clinicopathologic parameters in patients with CRC. The above data were indicated that lncRNA-KAT7 might be involved in the occurrence and progression of CRC. We divided the 140 patients with CRC into a high lncRNA-KAT7 tumor expression group (n = 70) and a low expression group (n = 70) (Table 1). As shown in Table 1, the expression level of lncRNA-KAT7 in cancer tissues was associated with tumor differentiation (*P *= 0.034), lymph node metastasis (*P *= 0.042), tumor size (*P *= 0.011), tumor site (*P *= 0.027). The above data shows that lncRNA-KAT7 may be involved in the development of CRC.

### LncRNA-KAT7 is lowly expressed in CRC cells

The relative expression level of lncRNA-KAT7 in CRC cell lines was further detected in CRC cells (Fig. 1f). Particularly, the expression levels of lncRNA-KAT7 in all 6 CRC cell lines (HCT116, SW620, LoVo, SW480, DLD1 and LS174T) are lower than that in the normal human colon tissue cells (CCD-18Co). The expression level of lncRNA-KAT7 in CRC cells corresponds to the level of histological outcomes. We chose HCT116 and DLD1 with relative low expression level of lncRNA-KAT7, for further study to assess the potential biological function of lncRNA-KAT7 in CRC.

### Overexpression of lncRNA-KAT7 inhibited the proliferation, migration and invasion of CRC cells

**Fig. 2:**
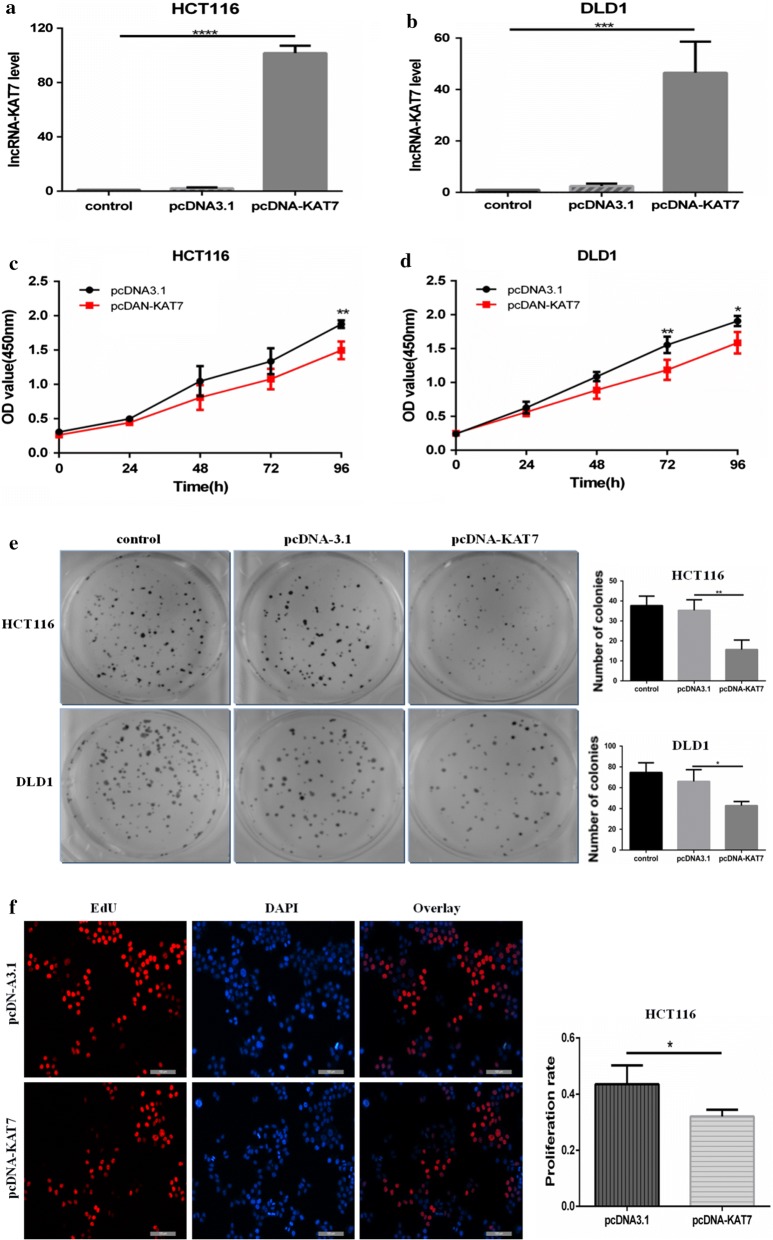
Overexpression of lncRNA-KAT7 inhibited the proliferation, colony formation of HCT116 and DLD1 cells. **a** HCT116 cells were stably transfected with lncRNA-KAT7 expression plasmid, and the efficiency of lncRNA-KAT7 overexpression was verified by real-time PCR, with the fold change of nearly 90 times. **b** DLD1 cells were stably transfected with lncRNA-KAT7 expression plasmid, and the efficiency of lncRNA-KAT7 overexpression was verified by real-time PCR, with the fold change of nearly 50 times. **c** CCK-8 assay showed that the proliferation rate of lncRNA-KAT7 overexpressed HCT116 cells was reduced. **d** CCK-8 assay showed that the proliferation rate of lncRNA-KAT7 overexpressed DLD1 cells was reduced. **e** Colony formation assay showed that overexpression of lncRNA-KAT7 reduced the colony formation of HCT116 and DLD1 cells. **f** EdU assay showed that the proliferation rate of lncRNA-KAT7 overexpressed HCT116 cells was reduced. *P < 0.05, **P < 0.01, ***P < 0.001

**Fig. 3:**
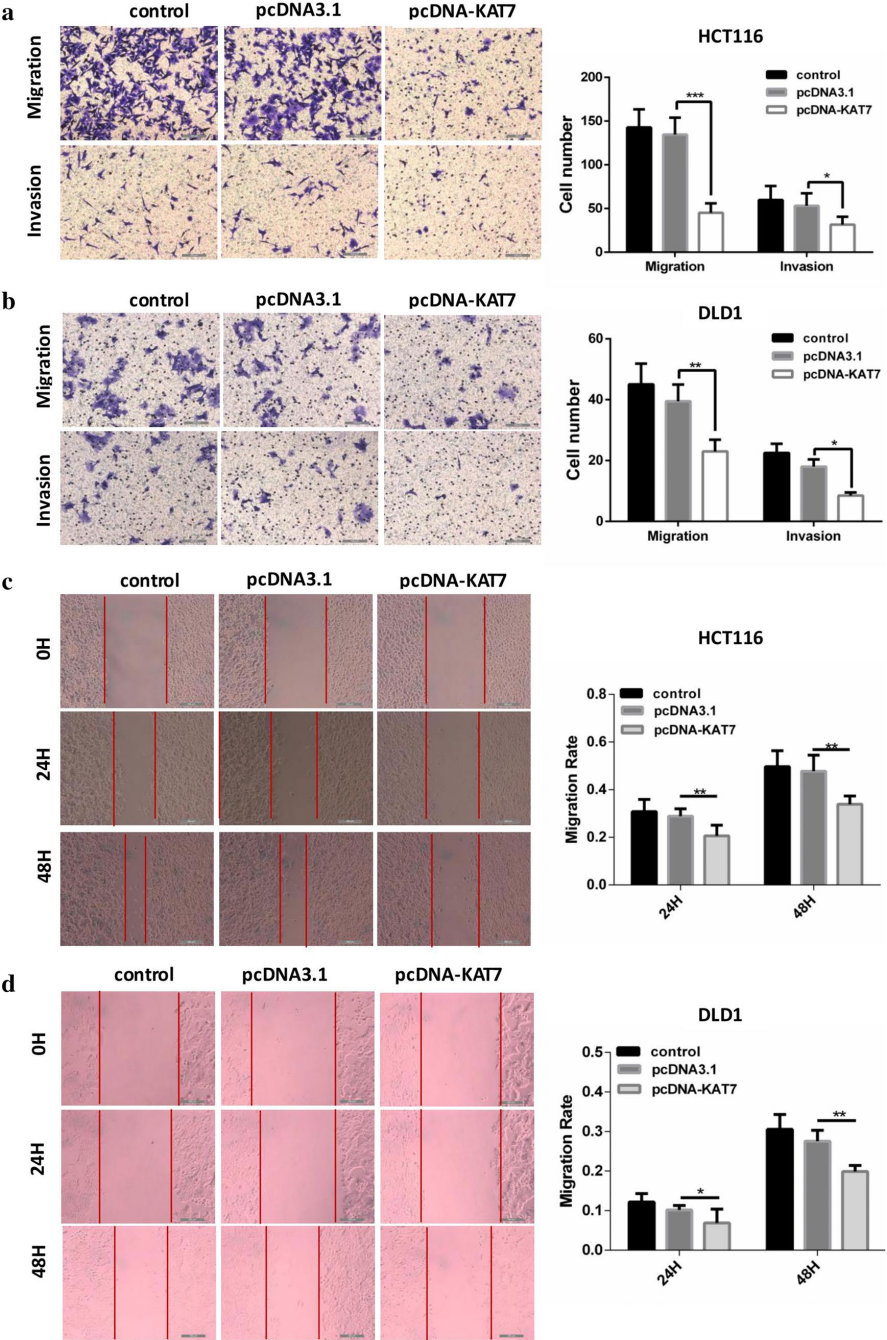
Overexpression of lncRNA-KAT7 inhibited the migration and invasion of HCT116 and DLD1 cells. **a** Transwell migration (upper) and invasion (lower) assays showed that overexpression of lncRNA-KAT7 could decrease the migration and invasion of HCT116 cells (Magnification × 200). **b** Transwell migration (upper) and invasion (lower) assays showed that overexpression of lncRNA-KAT7 could decrease the migration and invasion of DLD1 cells (Magnification × 200). **c** Wound healing assay showed that overexpression of lncRNA-KAT7 could inhibit the mobility of HCT116 cells (Magnification × 100). **d** Wound healing assay showed that overexpression of lncRNA-KAT7 could inhibit the mobility of DLD1 cells (Magnification × 100). *P < 0.05, **P < 0.01, ***P < 0.001

To elucidate the role of lncRNA-KAT7 in CRC progression, we have up-regulated the expression of lncRNA-KAT7 in HCT116 and DLD1 cells by using stably transfection. HCT116 and DLD1 cells were stably transfected with lncRNA-KAT7 expression plasmid, and the efficiency of lncRNA-KAT7 overexpression was verified by real-time PCR, with the change of approximately 90-fold and 50-fold, respectively (Fig. 2a, b). Our results showed that when lncRNA-KAT7 was overexpressed, the proliferation and colony forming abilities of HCT116 and DLD1 cells were inhibited compared to negative control cells (Fig. 2c–f). In transwell migration and invasion assays, overexpression of lncRNA-KAT7 attenuated the migratory and invasive abilities of HCT116 and DLD1 cells (Fig. 3a, b). In wound healing assays, lncRNA-KAT7 overexpression reduced the wound healing abilities of HCT116 and DLD1 cells (Fig. 3c, d). The above in vitro experiments performed in the two CRC cell lines suggested that lncRNA-KAT7 overexpression may suppress the malignant phenotypes of CRC cell lines, which was in correspondence with the clinical findings.

### Overexpression of lncRNA-KAT7 inhibited tumor growth in vivo

**Fig. 4:**
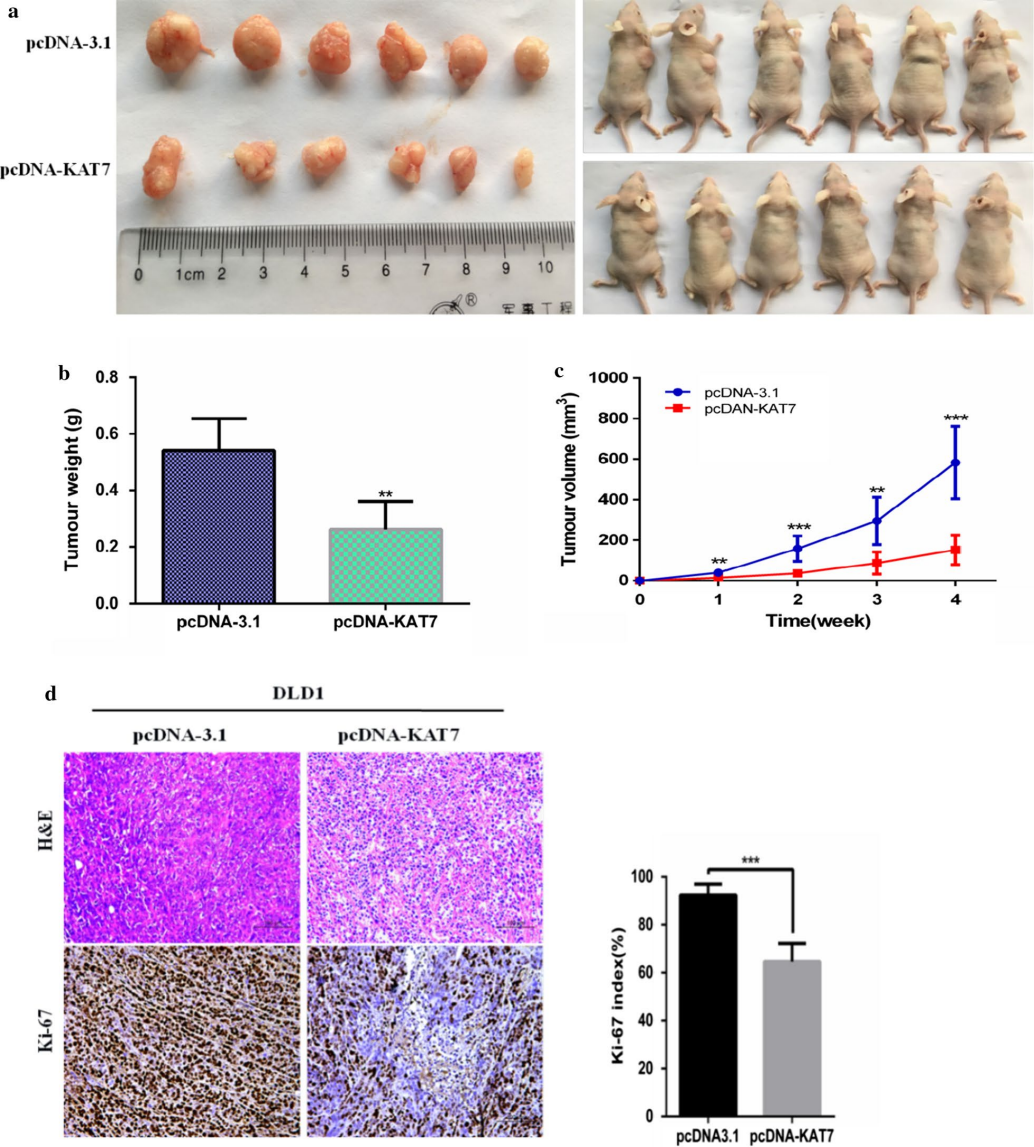
Over-expressed lncRNA-KAT7 inhibits the growth of CRC cells in vivo. **a** Up-regulation of lncRNA-KAT7 expression may inhibit the formation of subcutaneous tumor in a nude mouse model. DLD1/pcDNA3.1 and DLD1/pcDNA-KAT7 cells were inoculated subcutaneously on the back of nude mice. **b** DLD1/pcDNA-KAT7 cells were analyzed for the influence of KAT7 on tumor growth and weights in vivo, compared to DLD1/pcDNA3.1 group. **c** Comparison was made between the pcDNA3.1 group and the pcDNA-KAT7 group weekly using t-tests. **d** The tumor sections were subjected to H&E and IHC staining using antibodies against Ki-67 and the Ki-67 index was calculated as the number of Ki-67 positive cells divided by the number of total cells × 100%. Error bars indicate the mean ± SD of 6 different fields. Scale bars = 100 μm. **P < 0.01;***P < 0.001

To confirm effect of lncRNA-KAT7 on tumor proliferation in vivo, DLD1/pcDNA3.1 and DLD1/pcDNA-KAT7 cells were subcutaneously inoculated into the back of nude mice. Tumor size was measured over time. Mice in the DLD1/pcDNA-KAT7 group developed smaller tumors than those in the DLD1/pcDNA3.1 group (p < 0.01) (Fig. 4a–c). IHC assays confirmed that the Ki-67 proliferation index in the DLD1/pcDNA-KAT7–xenografted tumors was lower than that in DLD1/pcDNA3.1–xenografted tumors (Fig. 4c, d).

### LncRNA-KAT7 regulates the expression of proliferation, invasion and metastasis-associated protein in CRC

**Fig. 5:**
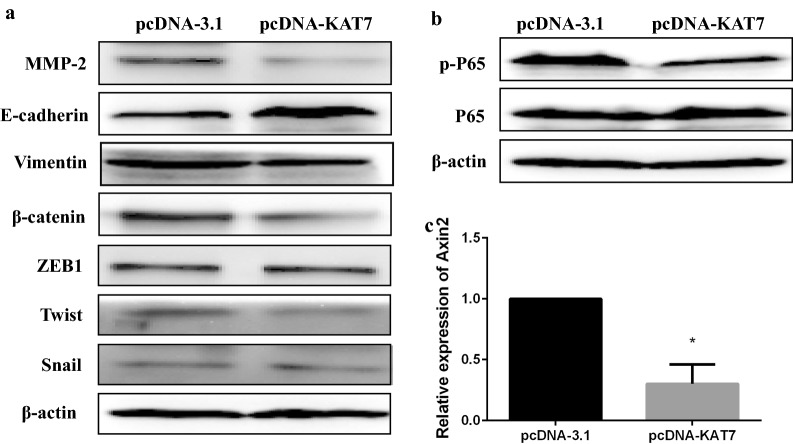
lncRNA-KAT7 regulates the expression of proliferation, migration and invasion-related proteins in CRC. **a** Protein expression levels of epithelial-mesenchymal transition (EMT)-related proteins were determined in HCT116 cells following lncRNA-KAT7 overexpression. **b** Protein expression levels of phosphorylated NF-κB P65 and NF-κB P65 were determined in HCT116 cells following lncRNA-KAT7 overexpression. **c** Relative expression of Axin2 was determined in HCT116 cells following lncRNA-KAT7 overexpression. *P < 0.05, Two-side Student’s t-test; n = 3

EMT is one of the classical pathways for metastasis of tumor cells, and deletion of E-cadherin and increase of Vimentin are the basic events for the formation of EMT [20–23]. Matrix metalloproteinases MMP-2 promotes the invasion and migration of tumor cells. To further explore the underlying role of lncRNA-KAT7 in CRC cancer biology, we detected the expression of EMT-related proteins by western blot. LncRNA-KAT7 expression increased the expression of E-cadherin in HCT116 cells and decreased the expression of Vimentin, MMP-2 and β-catenin, Twist (Fig. 5a), and did not affect the protein expression of ZEB1 and Snail [20–23].

Wnt/β-catenin and NF-κB signaling pathway play important roles in regulating the proliferation and migration of tumor cells, [26, 27]. Up-regulating of lncRNA-KAT7 attenuated the expression of phosphorylated NF-κB P65 in HCT116 cells (Fig. 5b), and the mRNA expression level of AXIN2, a β-catenin-target gene (Fig. 5c). Therefore, it was suggested that lncRNA-KAT7 can induce the proliferation, migration and invasion by regulating the expression of these proteins.

## Discussion

Long non-coding RNAs (long ncRNAs, lncRNA) are defined as transcripts longer than 200 nucleotides that are not translated into protein. This somewhat arbitrary limit distinguishes long ncRNAs from small non-coding RNAs such as microRNAs (miRNAs), small interfering RNAs (siRNAs), Piwi-interacting RNAs (piRNAs), small nucleolar RNAs (snoRNAs), and other short RNAs. Long intervening/intergenic noncoding RNAs (lincRNAs) are sequences of lncRNA which do not overlap protein-coding genes. Non-coding genomic portion accounts for 90% of mammalian whole genome [13]. LncRNA is a novel functional regulatory element that regulates gene expression and has undergone a transition from “biological waste, noise” to “biologically important regulators” [11]. The abnormal expression of lncRNA in tumors is closely related to the pathogenesis of tumors. However, the number of lncRNAs in CRC is still limited, and the molecular mechanism of the specific role is still not clear. It needs to be further improved. Metastasis results in approximately 90% of cancer-related mortality [28]. Although the mortality rate of CRC has declined in recent years, the prognosis of patients with metastatic CRC remains poor. Studies on lncRNAs in CRC may provide new insights into the development of CRC.

LncRNA has become a research hotspot and has attracted great interest from researchers around the world. Many lncRNAs display tissue and cell type-specific expression patterns at low expression levels and play a key regulatory role in biological processes. For example, Ye et al. [15] found that a novel lnc-GNAT1-1 is low-expressed in CRC and acts as a tumor suppressor by regulating the RKIP-NF-κB-Snail pathway. Zhou et al. [29] evaluated the potential of several lncRNAs as diagnostic markers for gastric cancer and finally demonstrated that plasma H19 may serve as a potential diagnostic biomarker for gastric cancer, especially in early-stage patients. It was reported that lncRNA FOXF1-AS1 is a possible cancer suppressor and its expression is decreased in gastric cancer tissues and lung cancer tissues. Miao et al. found that FOXF1-AS1 regulates E-cadherin and Vimentin in tumor metastasis in non-small cell lung cancer. Our previous study data have reported that lncRNA-KAT7 was low expressed in CRC cancer tissues and cells and negatively correlated with poor pathological characteristics such as tumor differentiation, tumor size, and lymph node metastasis in CRC patients. These studies suggest that lncRNA-KAT7 plays a role in the progression of CRC. Recently, many studies have focused on lncRNAs as potential stable and non-invasive tumor markers for cancer diagnosis and prognosis. Next, we will continue to complete the statistics of lncRNA-KAT7 expression level in CRC plasma and the pathological characteristics of the CRC patients, further supporting this hypothesis.

In vitro cell experiments showed that overexpression of lncRNA-KAT7 inhibited the proliferation, migration and invasion of CRC cells. In addition, to evaluate the effect of lncRNA-KAT7 on the growth of CRC tumors in vivo, we subcutaneously injected DLD1 cells overexpressing KAT7 into the back of nude mice, and the growth of the xenograft primary tumors was monitored. As shown in Fig. 3, xenograft tumors were generated at the injection site 10 days later. During 4 weeks of observation period, tumor growth in the KAT7 group was significantly slower than in the control group. Therefore, the tumor volume of the KAT7 group was significantly smaller than that of the control group. Immunohistochemical staining showed that Ki-67 index was lower in the KAT7 group than that in the control group (Fig. 3d). Low Ki-67 index demonstrated weak cell proliferation [6]. These data indicate that lncRNA-KAT7 plays a key role in the growth of CRC in vivo.

Although the specific mechanisms of lncRNAs involved in cancer biology have not been fully elucidated, a large number of studies have demonstrated that lncRNAs mainly regulate gene expression by interacting with proteins [30]. For example, HOTAIR, which is highly expressed in CRC stem cells, regulates the expression of E-cadherin, Vimentin, and N-cadherin in EMT-related molecules [31]. H19 was proved to be a novel regulator of EMT in CRC cells, interfering with the expression of H19 can significantly inhibit the expression of mesenchymal core marker gene vimentin, ZEB1 and ZEB2 in CRC cells [21]. Overexpression of lncRNA MEG3 in CRC can affect MMP-2 and MMP-9 expression to inhibit cell invasion and migration ability [11]. Deletion of E-cadherin and upregulation of vimentin are the basic events for the formation of EMT, which is one of the classical pathways for tumor cell metastasis. In this study, lncRNA-KAT7 significantly promoted the expression of E-cadherin, and inhibited the expression of Vimentin, β-catenin and Twist. Additionally, MMP2, an important factor for cell invasion, was downregulated by lncRNA-KAT7 in CRC. Therefore, lncRNA-KAT7 plays the important role in the migration and invasion by regulating EMT-related genes.

As one of the most studied transcription factors, NF-κB can regulate a variety of cellular processes in cancer, including proliferation, migration and invasion, angiogenesis, and chemotherapy resistance [27, 32]. In this study, lncRNA-KAT overexpression significantly suppressed the phosphorylation of NF-κB p65 in HCT116 cells. The expression of proliferation-related β-catenin was relatively weakened, and so did the expression level of Twist, a Wnt/β-catenin signal-related molecular. These data suggest that lncRNA-KAT7 may inhibit the proliferation and metastasis of CRC cells by modulating the expression of EMT-related proteins through regulating NF-κB signal pathway and Wnt/β-catenin signal pathway.

We have demonstrated that overexpression of lncRNA-KAT7 can inhibit the malignant phenotype of CRC cells, and low expression of lncRNA-KAT7 may lead to a more aggressive CRC cell phenotype. It was confirmed that lncRNA-KAT7 acts as a new lncRNA to inhibit cell proliferation and metastasis in CRC to exert tumor suppressing activity. However, this study also has some shortcomings. It is necessary to further verify the molecular mechanism of lncRNA-KAT7 involving in CRC invasion and metastasis. The in-depth study of these functional experiments and molecular mechanisms will provide new ideas, new strategies and new targets for the diagnosis and treatment of CRC.

## Conclusion

Generally, we have identified a novel lncRNA, lncRNA-KAT7 is under-expressed in CRC tissues and negatively correlated with tumor differentiation, tumor size, and lymph node metastasis in CRC patients. Up-regulation of lncRNA-KAT7 expression in CRC cells not only inhibited cell proliferation, migration and invasion in vitro, but also inhibited tumor growth in vivo. Therefore lncRNA-KAT7 is likely to exert tumor suppressor activity in the development of CRC. Our findings will provide new biomarkers and therapeutic candidates for CRC.

## Abbreviations

CRC: colorectal cancer
lncRNAs: long non-coding RNAs
lncRNA-KAT7: long non-coding RNA-KAT7
SNP: single nucleotide polymorphisms
VEGFA: vascular endothelial growth factor A
SFPQ: splicing factor proline and glutaminerich
RKIP: Raf-kinase inhabiory protein
ZEB1: Zinc finger E-box Binding homeobox 1
